# Rapid screening of MDR-TB using molecular Line Probe Assay is feasible in Uganda

**DOI:** 10.1186/1471-2334-10-41

**Published:** 2010-02-26

**Authors:** Heidi Albert, Freddie Bwanga, Sheena Mukkada, Barnabas Nyesiga, Julius Patrick Ademun, George Lukyamuzi, Melles Haile, Sven Hoffner, Moses Joloba, Richard O'Brien

**Affiliations:** 1 Foundation for Innovative New Diagnostics (FIND), Kampala, Uganda; 2 Department of Medical Microbiology, Makerere University, Kampala, Uganda; 3 National Tuberculosis Reference Laboratory, Wandegeya, Kampala, Uganda; 4 Department of Bacteriology, Swedish Institute for Infectious Disease Control, Solna, Sweden; 5 Foundation for Innovative New Diagnostics, Geneva, Switzerland

## Abstract

**Background:**

About 500 new smear-positive Multidrug-resistant tuberculosis (MDR-TB) cases are estimated to occur per year in Uganda. In 2008 in Kampala, MDR-TB prevalence was reported as 1.0% and 12.3% in new and previously treated TB cases respectively. Line probe assays (LPAs) have been recently approved for use in low income settings and can be used to screen smear-positive sputum specimens for resistance to rifampicin and isoniazid in 1-2 days.

**Methods:**

We assessed the performance of a commercial line probe assay (Genotype MTBDR*plus*) for rapid detection of rifampicin and isoniazid resistance directly on smear-positive sputum specimens from 118 previously treated TB patients in a reference laboratory in Kampala, Uganda. Results were compared with MGIT 960 liquid culture and drug susceptibility testing (DST). LPA testing was also performed in parallel in a University laboratory to assess the reproducibility of results.

**Results:**

Overall, 95.8% of smear-positive specimens gave interpretable results within 1-2 days using LPA. Sensitivity, specificity, positive and negative predictive values were 100.0%, 96.1%, 83.3% and 100.0% for detection of rifampicin resistance; 80.8%, 100.0%, 100.0% and 93.0% for detection of isoniazid resistance; and 92.3%, 96.2%, 80.0% and 98.7% for detection of multidrug-resistance compared with conventional results. Reproducibility of LPA results was very high with 98.1% concordance of results between the two laboratories.

**Conclusions:**

LPA is an appropriate tool for rapid screening for MDR-TB in Uganda and has the potential to substantially reduce the turnaround time of DST results. Careful attention must be paid to training, supervision and adherence to stringent laboratory protocols to ensure high quality results during routine implementation.

## Background

Uganda had an estimated incidence of all forms of TB of 330 per 100, 000 population in 2007, of which 136 per 100 000 were sputum smear positive. 0.5% of new TB cases and 4.4% of previously treated cases were multidrug resistant in 1997 [1]. However a recent drug resistance survey performed in Kampala reported prevalence of any drug resistance as 12.7% (63/497) and 31.6% (18/57), and MDR-TB prevalence of 1.0% (5/497) and 12.3% (7/57) in new and previously treated smear-positive TB cases respectively [2]. About 500 new smear-positive MDR-TB cases are estimated to occur per year in the country [1].

Recently the World Health Organisation (WHO) recommended the use of molecular line probe assays (LPAs) for rapid screening of MDR-TB in low and middle income settings [3]. LPAs use multiplex polymerase chain reaction (PCR) amplification and reverse hybridization to identify *M. tuberculosis *complex and mutations to genes associated with rifampicin and isoniazid resistance. LPA can be performed directly from acid fast bacilli (AFB) smear-positive sputum, or from culture isolates, and provide results in 1-2 days. A recent systematic review concluded that line probe assays are highly sensitive and specific for detection of rifampicin resistance (≥97% and ≥99%) and isoniazid resistance (≥90% and ≥99%) on culture isolates and smear-positive sputum. Overall agreement with conventional DST for detection of MDR-TB was 99% [4].

It is estimated that only 5% of patients with MDR-TB are currently detected worldwide. Lack of laboratory capacity in high TB burden countries, notably in sub-Saharan Africa, is a barrier to control of drug resistant TB. Conventional drug susceptibility testing (DST) is a slow process and can take 2-4 months or longer, during which time a patient is often treated according to the standard regimen for drug-susceptible TB. The resultant delay in proper treatment may adversely affect treatment outcome and contribute to the transmission of drug-resistant disease and amplification of drug resistance.

Furthermore, due to financial, infrastructural and human resource requirements, widespread implementation of culture-based DST may be challenging in such settings. Specimen transport and specimen contamination issues may also present further challenges [5]. Thus implementation of rapid molecular methods for detecting drug-resistant TB may be a viable alternative to culture-based DST in Uganda.

In response to the need to scale up access to TB diagnostic services, UNITAID has recently funded a project to introduce new TB diagnostics in selected low income countries. Project partners include WHO - Global Laboratory Initiative (GLI), FIND and the Stop TB Partnership's Global Drug Facility (GDF). This project has recently been expanded to include 27 countries, of which 12 are in sub-Saharan Africa, including Uganda.

We report on a local validation of rapid molecular testing using line probe assays for screening MDR-TB in Uganda, which was carried out as the first step in implementation of the LPA technology in the country.

## Methods

### Study setting

Previously treated pulmonary tuberculosis patients were enrolled at the Tuberculosis Unit at Mulago National Referral Hospital, Kampala. The line probe assay (LPA) testing and MGIT culture and DST were carried out at the Tuberculosis Research Laboratory operated by the Foundation for Innovative New Diagnostics (FIND) based at the National Tuberculosis Reference Laboratory (NTRL) in Kampala, Uganda. Inter-laboratory LPA testing was performed at the Department of Medical Microbiology, Makerere University and specimen decontamination and primary culture was carried out at the NTRL. The study was approved by Makerere University and Mulago Hospital Ethics committees.

### Specimen collection and processing

Smear-positive sputum was collected from patients at risk of MDR-TB at Mulago Hospital Previously treated TB suspects attending the Mulago National Referral Hospital TB Unit were consecutively screened for acid fast bacilli (AFBs) using Ziehl-Neelsen (ZN) smear microscopy, as part of a larger study investigating a number of rapid drug susceptibility test methods. All consenting smear positive patients were enrolled. Two or three sputum samples (spot or morning) were collected per patient in 50 ml sterile conical centrifuge tubes.

All manipulations with potentially infectious clinical specimens were performed in a Class II safety cabinet in a BSL 2 or 3 Laboratory. Sputum was processed with the *N*-acetyl-*L*-cysteine-sodium hydroxide (NaOH) method (NaOH final concentration, 1.5%).

Any processed specimen remaining was stored at 2-8°C for the duration of the study to allow for re-testing of specimens giving discrepant results.

### Conventional laboratory testing

Sputum specimens submitted for smear, culture and DST were processed using N-acetyl-cysteine-sodium hydroxide (NALC-NaOH) decontamination (NaOH final concentration, 1.5%) [6]. Following centrifugation, the pellet in each tube was suspended in 2.5 ml of phosphate buffer pH 6.8. Processed sediments from the same patient were pooled and mixed thoroughly. A concentrated auramine smear was prepared and examined under × 400 magnification using a fluorescence microscope and graded according to WHO/IUATLD guidelines [7]. Samples were cultured using the BACTEC MGIT 960 system (Becton Dickinson Microbiology Systems, Cockeysville, MD, USA) and Lowenstein-Jensen solid medium. Primary isolates were stored at -80°C. For MGIT DST, frozen isolates were cultured using MGIT 960 system and confirmed as *M. tuberculosis *complex using Capilia TB Neo (TAUNS Corporation, Japan) and checked for contamination by growth on blood agar medium for 48 hours at 37°C prior to setting up DST for isoniazid and rifampicin according to manufacturer's instructions (0.1 μg/ml isoniazid and 1 μg/ml rifampicin).

Any processed specimen remaining after initial cultures was stored at -20°C for the duration of the study to allow for re-testing of specimens in case of invalid results.

### Line probe assay (LPA)

LPA testing was performed in three separate rooms, according to WHO recommendations [3]. DNA extraction was performed in the BSL3 laboratory, master mix preparation in a second room, and PCR and hybridisation were performed in a third laboratory. Five hundred microlitres of processed sediment was used to perform the Genotype MTBDR*plus *(Hain Lifescience GmbH) assay, according to the manufacturer's instructions [8]. LPA was performed without knowledge of conventional DST results. Residual processed specimens were refrigerated at 2-8°C overnight after DNA extraction to allow repeat testing if required. In parallel, LPA testing was also performed on processed sediments in a blinded fashion at Makerere University Department of Medicine molecular biology laboratory.

### Repeat testing and discrepant analysis

LPA testing on samples with invalid results were repeated using the stored residual extracted DNA. To consider a band valid for study purposes, the band intensity had to be equal or greater than the AC band (according to the product insert).

### Inter-laboratory comparison

Testing at the University laboratory was completely independent of testing in the FIND-NTRL laboratory and was performed without knowledge of conventional DST results. Results from the University laboratory were reported and included in the data analysis after completion of all testing.

### Data analysis

The sensitivity, specificity, PPV, NPV and overall accuracy of LPA results were compared to the conventional MGIT DST results for rifampicin, isoniazid, multidrug resistance and the ability of rifampicin resistance alone to predict MDR. An analysis of banding patterns associated with rifampicin and isoniazid resistance in MDR-TB and non MDR-TB strains was performed.

Statistical tests were performed using Intercooled STATA 8.0 software (Statacorp LP, College Station, TX, USA) and Microsoft Excel 7.0 (Microsoft Corporation). Results were considered significant at p < 0.05.

## Results

Overall, 118 pooled smear-positive sputum specimens (118 patients) were tested by LPA. Of these, 53 (44.9%) were 3+ AFB smear-positive, 31 (26.3%) were 2+ smear-positive, 30 (25.4%) were 1+ smear-positive and 4 (3.4%) were very low positive (6-8 AFBs).

Conventional DST results were only available for 92 specimens. The remainder was not available due to lack of growth from the frozen primary isolate (15), isolation of non-tuberculous mycobacteria (1), contamination (2) or unavailability of frozen isolate for MGIT DST (8).

### Interpretation of LPA results

A total of 113/118 (95.8%) specimens gave interpretable LPA results overall, including results of repeat testing which was performed in 23 cases. The causes of repeat testing included no TUB band for 16 specimens (a single batch of 6 specimens also had lack of AC band on the negative control), *rpoB *band being very faint or absent (2), or faint or indistinct bands (5).

The proportion of invalid results (after repeat testing) was related to smear status, with 1.9% (1/53), 3.2% (1/31), 6.7% (2/30) and 25.0% (1/4) of results being invalid for 3+, 2+, 1+ and scanty smear-positive sputum specimens respectively.

A summary of LPA results, including results of repeat testing where performed, is shown in Table 1. Five strains were initially considered to be sensitive during the initial interpretation by the technologists, when strictly following the product insert, which states: "only those bands whose intensities are about as strong as or stronger than that of the Amplification control zone are to be considered". However, when re-checked by the supervisor, these strains were considered to be resistant since mutation bands were present, although they were somewhat weaker than the amplification control. These strains were also considered to be resistant by the University laboratory (read independently from the research laboratory). All 5 strains were later confirmed to be drug resistant by MGIT DST.

**Table 1:** Performance of line probe assay (LPA) in smear-positive sputum specimens compared with conventional drug susceptibility testing (MGIT DST)

		MGIT DST				
		**INH^R ^RIF^R^**	**INH^R ^RIF^S^**	**INH^S ^RIF^R^**	**INH^S ^RIF^S^**	No result*

**LPA**	**INH^R ^RIF^R^**	12	3	0	0	4
	
	**INH^R ^RIF^S^**	0	6	0	0	0
	
	**INH^S ^RIF^R^**	1	0	2	1	2
	
	**INH^S ^RIF^S^**	0	4	0	63	15
	
	**Indeterminate**	0	0	0	0	5

LPA, line probe assay

MGIT DST, Mycobacterial Growth Indicator Tube

RIF^S^, rifampicin susceptible; RIF^R^, rifampicin resistant

INH^S^, isoniazid susceptible; INH^R^, isoniazid resistant

* No result was due to lack of growth from the frozen primary isolate (15), isolation of non-tuberculous mycobacteria (1), contamination (2) or unavailability of frozen isolate for MGIT DST (8).

Indeterminate results included are those remaining without an interpretable result after repeat testing.

Performance parameters for LPA were calculated by comparison with conventional culture and DST results (Table 2). The sensitivity for detection of rifampicin, isoniazid and multidrug resistance was 100.0%, 80.8% and 92.3% respectively. Specificity for detection of rifampicin, isoniazid and multidrug resistance was 96.1%, 100.0% and 96.2% respectively. When rifampicin resistance alone was used as an indicator for MDR-TB, the agreement remained very high, with 96.7% of results correctly predicting MDR.

**Table 2:** Performance of line probe assay (LPA) in detecting rifampicin, isoniazid and multidrug-resistance from smear-positive sputum specimens

	Rifampicin	Isoniazid	Multi-drug resistance	Rifampicin as predictor of MDR
No. resistant/No. susceptible strains	15/77	26/66	13/79	13/79

Sensitivity, % (95% CI)	100.0% (78.2 - 100.0)*	80.8% (60.6 - 93.4)	92.3% (64.0 - 99.8)	100.0% (75.3-100.0)*

Specificity, % (95% CI)	96.1% (89.0 - 99.2)	100.0% (94.5 - 100.0)*	96.2% (89.3 - 99.2)	96.2% (89.3 - 99.2)

Overall accuracy, % (95% CI)	96.7% (90.8 - 99.3)	94.6 (87.8 - 98.2)	95.7% (89.2 - 98.8)	96.7% (90.7 - 99.3)

PPV, % (95% CI)	83.3% (58.6 - 96.4)	100.0% (83.9 - 100.0) *	80.0% (51.9 - 95.7)	81.3% (54.3 - 96.0)

NPV, % (95% CI)	100.0% (95.1 - 100.0)	93.0% (84.3 - 97.7)	98.7% (92.9 - 100.0)	100.0% (95.3 - 100.0)*

* one-sided, 97.5% confidence interval

### Banding patterns

The patterns of mutations associated with rifampicin and isoniazid resistance in multidrug resistant and mono-resistant strains is shown in Table 3.

**Table 3:** Pattern of gene mutations detected by Genotype MTBDR*plus *assay in drug resistant *M. tuberculosis *strains

Gene	Band	Gene region or mutation	MDR strains* (n = 13)	RIF monoresistant strains* (n = 6)	INH monoresistant strains* (n = 13)**
*rpoB*					

	WT1	506-509			

	WT2	510-513		1	

	WT3	513-517	1	2	

	WT4	516-519		1	

	WT5	518-522			

	WT6	521-525			

	WT7	526-529	1	1	

	WT8	530-533	1	1	

	MUT1	D516V			

	MUT2A	H526Y			

	MUT2B	H526D	1		

	MUT3	S531L	9	1	

*katG*					

	WT	315	1		1

	MUT1	S315T1	9		7

	MUT2	S315T2			

*inhA*					

	WT1	-15/-16	1#		

	WT2	-8			

	MUT1	C15T	2		1

	MUT2	A16G			

	MUT3A	T8C			

	MUT3B	T8A			

* by conventional drug susceptibility testing (MGIT DST)

1 MDR strain had both *rpoB *WT3 and WT4 mutations, 1 MDR had both *rpoB *WT4 and WT7 mutations.

# this strain also had *katG *S315T1 mutation.

1 MDR strain did not have mutation in either *katG *or *inhA*.

** 4 INH mono-resistant strains had no mutations detected in *inhA *or *katG*.

### Inter-laboratory comparison

Of the 118 smear-positive specimens, 117 were tested by the University laboratory. Overall, 109 specimens gave interpretable results, with 20 MDR-TB and 89 non-MDR-TB results. 8 specimens gave indeterminate results. There was very high concordance (98.1%) between results obtained by FIND-NTRL laboratory and the University laboratory. Two specimens were reported as MDR-TB by the University laboratory but non MDR (rifampicin monoresistant) by the FIND-NTRL laboratory. One of these specimens was confirmed as MDR-TB by MGIT DST and the other specimen had no MGIT DST result available. Table 4 shows the comparison of results for the 88 specimens in which both LPA and MGIT DST results were available.

**Table 4:** Comparison of line probe assay (LPA) results from FIND-NTRL and University laboratory (specimens in which both LPA and MGIT DST results were available)

MGIT DST result	LPA results	Number of specimens
RIF^R ^INH^R ^(n = 12)	Lab 1 and Lab 2 MDR	11

	Lab 1 RIF^R ^INH^S^; Lab 2 MDR	1

RIF^S ^INH^R ^(n = 13)	Lab 1 and Lab 2 RIF^R ^INH^R^	3

	Lab 1 and Lab 2 RIF^S ^INH^R^	6

	Lab 1 and Lab 2 RIF^S ^INH^S^	4

RIF^R ^INH^S ^(n = 2)	Lab 1 and Lab 2 RIF^R ^INH^S^	2

RIF^R ^INH^S ^(n = 61)	Lab 1 and Lab 2 RIF^S ^INH^S^	59

	Lab 1 RIF^S ^INH^R^; Lab 2 RIF^S ^INH^S^	2

Total		88

Lab 1, FIND-NTRL laboratory

Lab 2, University laboratory

RIF^S^, rifampicin susceptible; RIF^R^, rifampicin resistant

INH^S^, isoniazid susceptible; INH^R^, isoniazid resistant

## Discussion

The performance of LPA directly from smear-positive sputum correlated very highly with culture and DST performed on MGIT 960. Overall, an acceptable proportion of valid results was obtained. Initial invalid results were largely due to insufficient DNA in two batches of tests which may be due to operator error in the extraction process. Repeat testing gave interpretable results in most cases. As previously reported, the proportion of invalid results was correlated with smear status, with much higher failure rates in very low smear-positive specimens [9].

As reported widely elsewhere, rifampicin resistance was highly associated with mutation in the 81 base pair region of the *rpoB *gene [10,11]. In this study it was most commonly associated with mutation in the region of *rpoB *530-533, mostly S531L mutation. This mutation was more frequently found in MDR strains than in rifampicin monoresistant strains. This is in common with findings in a recent South African study [9]. Isoniazid resistance was most commonly associated with *katG *S315T1 mutation, as is the case in many high TB burden countries, presumably related to ongoing transmission of these strains [12]. This was equally the case in MDR and INH monoresistant strains, although the overall number of strains was small. The performance of the LPA in this setting was similar to that reported previously, with high specificity for detection of rifampicin and isoniazid, high sensitivity for detection of rifampicin resistance, and somewhat lower sensitivity for isoniazid resistance [4]. One MDR strain in this study did not reveal mutations in the selected *katG *or *inhA *loci which are detected by this test. The strain was not further investigated as to the mutation(s) associated with INH resistance in this case. Studies from a number of countries have reported variability in the association of isoniazid resistance with mutations in *katG *or *inhA *[13].

Laboratory technologists performing the assay at the NTRL-FIND Laboratory had no previous experience of molecular diagnostics, and had undergone 4 days of LPA training immediately prior to starting the study. This may explain the fact that invalid results were initially obtained on some batches, and may be related to operator error during DNA extraction. However, performance overall in the validation study was very good, and comparable with results in other settings. In addition, results were highly reproducible with >98% of results in concordance with results of independent testing performed at the University laboratory.

Although in most cases the interpretation of banding patterns was very straightforward, in a few specimens weak mutation bands were present, which if strictly applying recommendations in the product insert would be considered as susceptible. However, we found the presence of weak mutation bands was associated with drug resistance in all cases in this study. This needs to be further confirmed in testing of a larger number of specimens.

The technologists involved in this study considered 4 days training to be sufficient. However as evidenced by the initial problem with invalid results, as well as difficulty interpreting weak mutation bands and dealing with contamination, a longer supervised period during early implementation of the technology is advisable, and the availability of an experienced molecular biologist in person, or at the very least by regular email contact, is critical in troubleshooting problems especially during the initial stages.

Issues to be considered during implementation of LPA in high TB burden countries include supply of consumables and reagents not provided as part of the kit, such as pipette tips and molecular grade water. Furthermore, the necessary infrastructure for performing LPA should be considered prior to implementation - a minimum of three separate rooms is recommended to minimise the risk of contamination. Restricted access to the molecular laboratories and strict adherence to standard operating procedures are necessary to reduce the risk of amplicon contamination. During this study, we experienced a single minor contamination episode at the FIND-NTRL laboratory involving splashing from one well to another during the hybridization process leading to contamination of the negative control strip, necessitating repeating the batch of testing.

The NTRL has established a specimen referral network which is being rolled out in at the regional level to enable transport of smear-positive specimens from MDR-TB suspects for DST. Currently DST is performed using MGIT 960, but this comes with a number of challenges related to the need for cold storage of specimens during transport and high level of contamination. Implementation of LPA is anticipated to lead to a more rapid turnaround time, and a higher proportion of valid results, as well as eliminating the need for cold storage (unless culture is also to be performed on the same specimens). With expansion of routine LPA testing, it will be critical to implement quality assurance procedures, and to provide access to technical support for laboratory staff, to ensure consistency of results.

LPAs are currently validated only for use directly from smear-positive specimens, although reasonable performance in a small sample of smear-negative specimens was demonstrated by Barnard and colleagues [9]. Although smear-positive TB cases are the most infectious [14], smear-negative TB in high HIV prevalent settings such as Uganda is responsible for substantial morbidity and mortality [15]. In Uganda, for example, sputum smear-positive cases represent only 41% of all new TB cases per year [1].

Ongoing research into improved DNA extraction methods may enable LPAs to be performed directly from smear-negative sputum in future. However the cost-effectiveness of routine testing of smear-negative specimens would have to be carefully evaluated since the majority of specimens will be negative in most settings.

LPA supplies are available in Uganda at a reduced price. As part of its role in the development and evaluation process of new diagnostic technologies, FIND has negotiated with the manufacturing partner to obtain significant price reductions for equipment and reagents for LPA testing for the public health sector in high burden countries in order to facilitate widespread access to WHO-approved technologies [16].

## Conclusions

LPA is an appropriate tool for rapid screening for MDR-TB in the Ugandan reference laboratory setting and has the potential to substantially reduce the turnaround time of DST results. However, WHO recommendations on infrastructure, training, quality assurance and other requirements should be followed to ensure high quality results. Good communication between laboratory and clinical personnel is critical to ensure rapid referral of specimens and receipt of results to enable the full benefit of rapid diagnostics to be realised. Furthermore, roll out of improved diagnostic technologies should happen in parallel with plans for increasing MDR-TB management capacity, as implementation of a rapid diagnostic such as LPA can only impact on patient care as part of a holistic approach to MDR-TB management.

## Competing interests

HA, PJA, GL, BN and ROB are employed by the Foundation for Innovative New Diagnostics (FIND). SM was based at FIND Uganda as an intern for the duration of this study. FB, MJ, MH and SH declare they have no competing interests.

## Authors' contributions

HA participated in study design, coordinated the project, performed data analysis and drafted the manuscript. FB participated in study design, coordinated specimen collection, performed testing at University laboratory and critically reviewed the manuscript. SM carried out LPA testing, participated in study coordination and data analysis and helped to draft the manuscript. NB carried out LPA testing and participated in study coordination. GL and PJA participated in LPA and carried out conventional laboratory testing. ROB and MJ conceived of the study, participated in study design and critically reviewed the manuscript. MH and SH participated in study design and critically reviewed the manuscript. All authors read and approved the final manuscript.

## Pre-publication history

The pre-publication history for this paper can be accessed here:

http://www.biomedcentral.com/1471-2334/10/41/prepub

## References

[B1] World Health Organisation Global tuberculosis control - epidemiology, strategy, financing WHO Report 2009 WHO/HTM/TB/2009.411 http://www.who.int/tb/publications/global_report/2009/en/index.html Accessed 19 November 2009

[B2] Lukoye D Cobelens F Ezati N Kirimunda S Adatu F Konde J Joloba M Anti-tuberculosis drug resistance in Kampala, Uganda, has not changed and shows no association with HIV infection The 27th Uganda Medical Laboratory Technology Association Scientific Conference, Kabale, Uganda 2009

[B3] World Health Organisation Policy Statement. Molecular Line Probe Assays for Rapid Screening of patients at risk of multidrug resistant tuberculosis (MDR-TB), 2008 http://www.who.int/tb/dots/laboratory/lpa_policy.pdf Accessed 18 November 2009

[B4] Ling D Zwerling A Pai M GenoType MTBDR assays for the diagnosis of multidrug-resistant tuberculosis: a meta-analysis Eur Respir J 2008 32 1165 1174 10.1183/09031936.00061808 18614561

[B5] World Health Organisation Use of Liquid TB culture and drug susceptibility testing (DST) in low- and medium- income settings 2007 http://www.who.int/tb/dots/laboratory/policy/en/index3.html Accessed 18 November 2009

[B6] Master RN Isenburg HD Mycobacteriology Clinical Microbiology Procedures Handbook. Section 3 1992 1 American Society for Microbiology, Washington DC

[B7] World Health Organisation WHO Laboratory Services in Tuberculosis Control. Part II: Microscopy. WHO/TB/98.258 1998 Geneva, Switzerland: WHO

[B8] Hain Lifescience Genotype® MTBDRplus product insert. Version 1 http://www.hain-lifescience.de/en/products/microbiology/mycobacteria/genotype-mtbdrplus.html

[B9] Barnard M Albert H Coetzee G O'Brien R Bosman ME Rapid molecular screening for multidrug-resistant tuberculosis in a high-volume public health laboratory in South Africa Am J Respir Crit Care Med 2008 177 7 787 792 10.1164/rccm.200709-1436OC 18202343

[B10] Telenti A Imboden P Marchesi F Cole S Colston MJ Matter L Schopfer K Bodmer T Detection of rifampicin-resistance mutations in *Mycobacterium tuberculosis* Lancet 1993 341 647 650 10.1016/0140-6736(93)90417-F 8095569

[B11] Van Rie A Warren R Mshanga I Jordaan A Spuy GD van der Richardson M Simpson J Gie RP Enarson DA Beyers N van Helden PD Victor TC Analysis for a limited number of gene codons can predict drug resistance of *Mycobacterium tuberculosis *in a high-incidence community J Clin Microbiol 2001 39 636 641 10.1128/JCM.39.2.636-641.2001 11158121 PMC87790

[B12] Mokrousov I Narvskaya O Otten T Limenschenko E Steklova L Vyshnevskiy B High prevalence of katG Ser315Thr substitution among isoniazid-resistant *Mycobacterium tuberculosis *isolates from Northwestern Russia, 1996-2001 Antimicr Agents Chem 2002 46 1417 1424 10.1128/AAC.46.5.1417-1424.2002 PMC127151 11959577

[B13] Baker LV Brown TJ Maxwell O Gibson AL Fang Z Yates MD Drobniewski FA Molecular analysis of isoniazid-resistant *Mycobacterium tuberculosis *isolates from England and Wales reveals the phylogenetic significance of the ahpC-46A polymorphism Antimicr Agents Chem 2005 49 1455 1464 10.1128/AAC.49.4.1455-1464.2005 PMC1068606 15793126

[B14] Behr MA Warren SA Salamon H Hopewell PC Ponce de Leon A Daley CL Small PM Transmission of *Mycobacterium tuberculosis *from patients smear negative for acid fast bacilli Lancet 1999 353 444 449 10.1016/S0140-6736(98)03406-0 9989714

[B15] Getahun H Harrington M O'Brien R Nunn P Diagnosis of smear-negative pulmonary tuberculosis in people with HIV infection or AIDS in resource-constrained settings: informing urgent policy changes Lancet 2007 369 9578 2042 2049 10.1016/S0140-6736(07)60284-0 17574096

[B16] Foundation for Innovative New Diagnostics FIND prices for GenoType^® ^MTBDRplus and Country List 2009 http://www.finddiagnostics.org/programs/find-negotiated-prices/mtbdrplus.html Accessed 2 February 2010

